# Molecular and phenotypic identification of bacterial species isolated from cows with mastitis from three regions of Poland

**DOI:** 10.1186/s12917-023-03869-w

**Published:** 2024-05-11

**Authors:** Anna Dobrut, Izabela Siemińska, Agnieszka Sroka-Oleksiak, Kamil Drożdż, Joanna Sobońska, Urszula Mroczkowska, Monika Brzychczy-Włoch

**Affiliations:** 1 Department of Molecular Medical Microbiology, Chair of Microbiology, Jagiellonian University Medical College, Krakow, Poland; 2 Institute of Veterinary Sciences, University Center of Veterinary Medicine JU-AU, University of Agriculture in Krakow, Krakow, Poland; 3 PetVet, Jedwabne, Poland

**Keywords:** *Streptococcus uberis*, *Streptococcus agalactiae*, *Staphylococcus aureus*, *Escherichia coli*, Mastitis, Bacterial identification, Epidemiological data, Polish cattle

## Abstract

**Background:**

Bovine mastitis is a widespread disease affecting dairy cattle worldwide and it generates substantial losses for dairy farmers. Mastitis may be caused by bacteria, fungi or algae. The most common species isolated from infected milk are, among others, *Streptococcus spp.*, *Escherichia coli*, *Staphylococcus aureus* and non-aureus staphylococci and mammaliicocci. The aim of this paper is to determine the frequency of occurrence of bacterial species in milk samples from cows with mastitis from three regions of Poland: the north-east, the south-west and the south. To this end 203 milk samples taken from cows with a clinical form (CM) of mastitis (n = 100) and healthy animals (n = 103) were examined, which included culture on an appropriate medium followed by molecular detection of *E. coli*, *S. aureus*, *Streptococcus agalactiae* and *Streptococcus uberis*, as one of the most common species isolated from mastitis milk.

**Results:**

The results obtained indicated that *S. uberis* was the most commonly cultivated CM species (38%, n = 38), followed by *S. aureus* (22%, n = 22), *E. coli* (21%, n = 21) and *S. agalactiae* (18%, n = 18). Similar frequencies in molecular methods were obtained for *S. uberis* (35.1%) and *S. aureus* (28.0%). The variation of sensitivity of both methods may be responsible for the differences in the *E. coli* (41.0%, p = 0.002) and *S. agalactiae* (5.0%, p = 0.004) detection rates. Significant differences in composition of species between three regions of Poland were noted for *E. coli* incidence (p < 0.001), in both the culture and molecular methods, but data obtained by the PCR method indicated that this species was the least common in north-eastern Poland, while the culture method showed that in north-eastern Poland *E. coli* was the most common species. Significant differences for the molecular method were also observed for *S. uberis* (p < 0.001) and *S. aureus* (p < 0.001). Both species were most common in southern and south-western Poland.

**Conclusions:**

The results obtained confirm the need to introduce rapid molecular tests for veterinary diagnostics, as well as providing important epidemiological data, to the best of our knowledge data on Polish cows in selected areas of Poland is lacking.

## Background

Bovine mastitis is a major disease that affects dairy cattle worldwide and generates great losses for the dairy industry [1]. It is estimated that the economic losses generated by clinical forms of mastitis may reach as much as 240 euros per cow per year [2, 3]. These financial losses tend to result from reduced volumes of milk, lower milk quality and the need to isolate sick animals, during which time they produce no milk and require treatment [1]. Mastitis may take the form of sub-clinical, clinical or chronic infections and this status may depend on the nature of the pathogen responsible and on the age, breed, immunological health and state of lactation of the animal [4, 5]. The clinical form of mastitis demonstrates visible and palpable symptoms such as redness of the udder, pain, swelling, fever or elevated temperature, a change in the appearance of milk and a reduction in the production of milk [4, 5]. The frequency of the clinical form of mastitis varies among regions and ranges from 12 to 30% [3]. The sub-clinical form generates the highest costs associated with difficulties in its detection due to the lack of visible symptoms. In turn, the chronic form is the least common but may lead to chronic mastitis [6].

Mastitis is generally caused by bacteria but also fungi and algae. The most common bacterial species responsible for mastitis are *Streptococcus uberis*, *Staphylococcus aureus*, *Streptococcus dysgalactiae, Streptococcus agalactiae* (group B streptococcus, GBS), *Non-aureus staphylococci and mammaliicocci* (NASM), *Corynebacterium bovis*, *Mycoplasma bovis*, *Escherichia coli* and *Klebsiella pneumoniae* [7, 8]. The frequency of mastitis in countries and regions varies depending on latitude, management conditions and diagnostic methodology [9].

The current gold standard in the diagnosis of bovine mastitis is a method based on the determining the somatic cell count (SCC) in quarter milk, 200.000 cells/ml being considered a critical value [10]. This method allows the rapid detection of mastitis in animals examined directly in the field, undoubtedly its most important advantage. California Mastitis Test (CMT) [11] is one of the methods for the rapid detection of high SCC in milk on field, however other on-farm culture methods exist and can be used on field [12, 13]. As a result, the infected animal may immediately be isolated from the healthy herd, thus thwarting the spread of the infection among the uninfected animals and helping avoid financial losses. This method, however, demonstrates a severe flaw: the inability of etiological factor identification. This lack of knowledge prevents veterinarians from introducing correct therapy which in turn leads to the increase of incorrect and unnecessary use of antibiotics [14], hence increase the adverse phenomenon leads to the increase of multidrug resistant bacteria. In response to this, scientists, supported by the biotechnology market, are therefore attempting to develop an alternative method, one that precisely, rapidly, and at limited costs allows the identification of the bacterial genus and species, and on the basis of which the administrated antibiotic would be selected precisely. Culture methods on an appropriated medium allows the identification of bacterial species, but waiting time for the results needs at least 24–72 h [15], and interpretation of the results may be ambiguous. Nucleic acid amplification techniques (NAATs) and serological methods are a promising alternative. As common advantages they share high selectivity and specificity, as the waiting time for the results is shorter than traditional culture. Another reliable and rapid technique to identify the most common microorganisms, which constantly develops is MALDI-TOF MS (Matrix-assisted laser desorption/ionization time-of-flight mass spectrometry). This method allows obtaining results in 24 h and the database are continuously expanded and improved with additional species [16]. A crucial limitation, however, is the inability to perform the tests outside a laboratory equipped with specialist equipment [17]. Immunochromatographic assay (lateral flow assay, LFA), which does not demonstrate the above limitation mentioned above is a promising alternative. This rapid test allows the detection of pathogens in biological samples within a few minutes. It also requires no specialized equipment; it is easy to perform and simple to interpret. Crucially, however, the lateral flow assay may be performed directly in the field. The low costs, in comparison with other molecular and serological methods, also make this technique more accessible, not only to veterinary surgeons but also to dairy farmers and cattle breeders [18].

The aim of this research was to characterize the most common species of bacteria that cause mastitis among cows in Poland. The results obtained for three regions in Poland (*voivodeship*s) may update the knowledge in the field of mastitis epidemiology among cows in these three regions Poland because, to the best of our knowledge, there is a lack of up-to-date data.

## Results

### Phenotypic and molecular identification

**Fig. 1:**
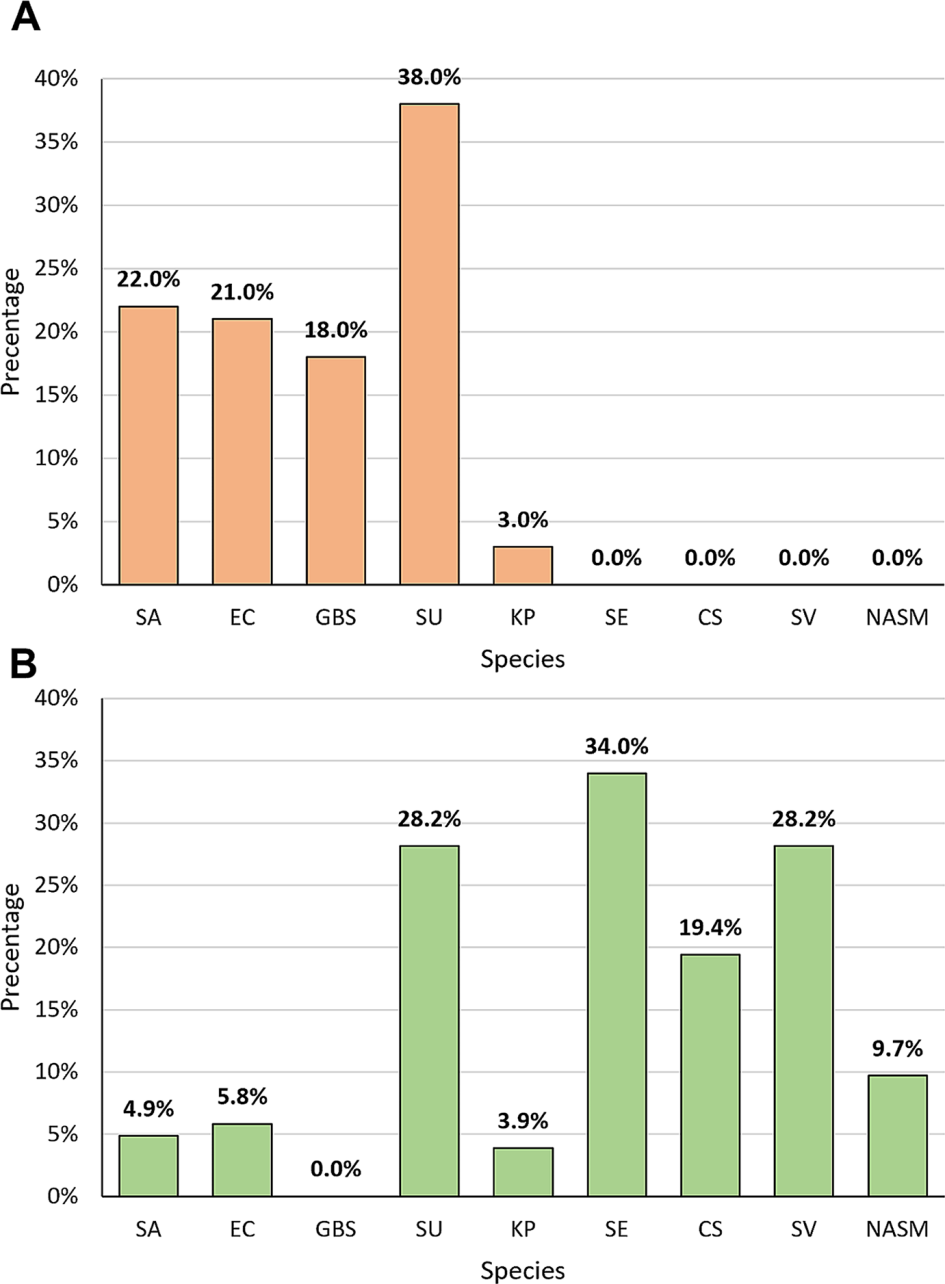
The percentage of bacterial species identified by using culture methods in bovine milk from cows with clinical mastitis (**A**) and from cows from the control group (**B**). Legend: SA – *Staphylococcus aureus*, EC – *Escherichia coli*, GBS – *Streptococcus agalactiae*, SU – *Streptococcus uberis*, KP – *Klebsiella pneumoniae*, SE – *Staphylococcus epidermidis*, CS – *Corynebacterium* spp., SV – *Viridians Group Streptococcus*, NASM – Non-aureus staphylococci and mammaliicocci (exc. *Staphylococcus epidermidis*)

In the course of identifying the most common species of bacteria in clinical samples from cows from three Polish voivodships, phenotypical and molecular identification were performed. Culture methods followed by phenotypic analyses indicated that the most common species in the studied CM samples was *S. uberis* (38.0%, n = 38), followed by *S. aureus* (22%, n = 22), *E. coli* (21%, n = 21), *S. agalactiae* (18%, n = 18), and *K. pneumoniae* (3%, n = 3) (Fig. 1A). A greater diversity in the composition of bacterial species was observed in the pool of samples from animals without clinical signs of mastitis. Bacterial composition identified in the pool of non-clinical samples included the following species: *Staphylococcus epidermidis* (34.0%, n = 35), *Viridans Group Streptococci* (SV) (28.2%, n = 29), *S. uberis* (28.2%, n = 29), *Corynebacterium* spp. (19.4%, n = 20), other NASM (excluding *S. epidermidis*) (9.7%, n = 10), *E. coli* (5.8%, n = 6), *S. aureus* (4.9%, n = 5), and *K. pneumoniae* (3.9%, n = 4) (Fig. 1B). According to National Mastitis Council (NMC) in the control group, 13 samples were classified as sub-clinical and 11 were classified as a contamination.

We also attempted to compare the accuracy between Columbia Blood Agar and Chromagar mastitis in detecting bacterial species that cause mastitis and we showed that Chromagar mastitis significantly increased the detectability of *S. agalactiae* (p < 0.001) more than Columbia Blood Agar. Detection levels for other species was comparable and statistically insignificant.

**Fig. 2:**
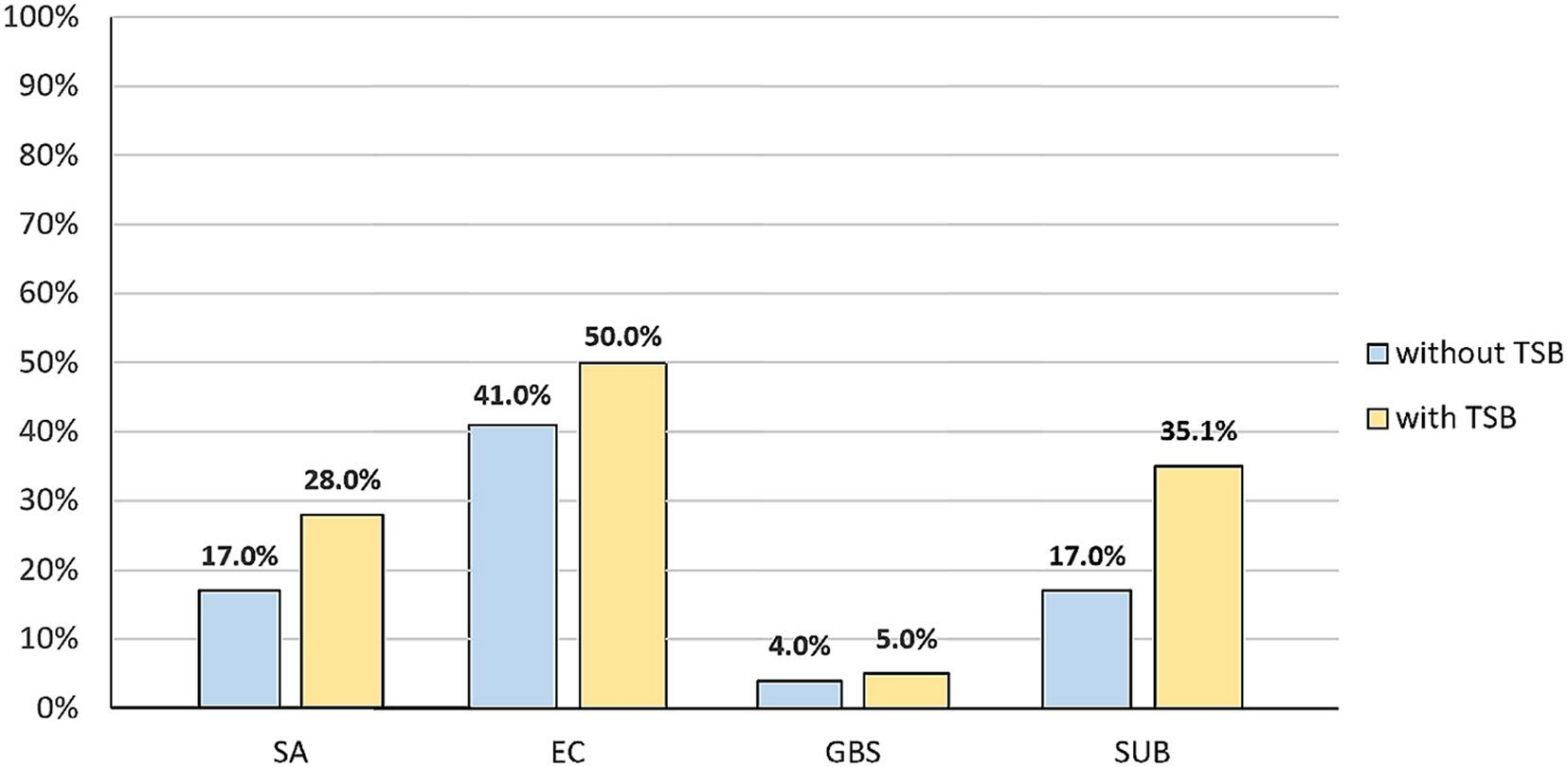
The results of a comparison between the two approaches of isolating the bacterial DNA from CM milk samples. Legend: without TSB – milk samples subjected to direct DNA isolation; with TSB – milk samples subjected to pre-incubation in TSB before further DNA isolation procedures; SA – *Staphylococcus aureus*, EC – *Escherichia coli*, GBS – *Streptococcus agalactiae*, SU – *Streptococcus uberis*

In the molecular analysis we began by comparing two methods of DNA isolation. In the first approach bacterial DNA was directly isolated and in the second samples were pre-incubated in TSB. The aim of this modification was to improve the amount of DNA obtained for further research. It was shown that pre-incubation in TSB improved sensitivity of the method for all species examined. Among the directly isolated CM pool of samples *S. aureus* was detected in 17% (n = 17) of the milk examined, while pre-incubation increased sensitivity by 11%. The introduction of the pre-incubation step for *E. coli* led to 9% growth in detection of the pathogen and the percentage of positive results increased from 41 to 50%. For *S. agalactiae* pre-incubation produced the detection of one more *S. agalactiae*-positive sample (to 5%). Most notable was the double growth observed for *S. uberis*, which allowed the detection of 35.1% of clinical milk samples and the differences were statistically significant (p = 0.005) (Fig. 2).

**Fig. 3:**
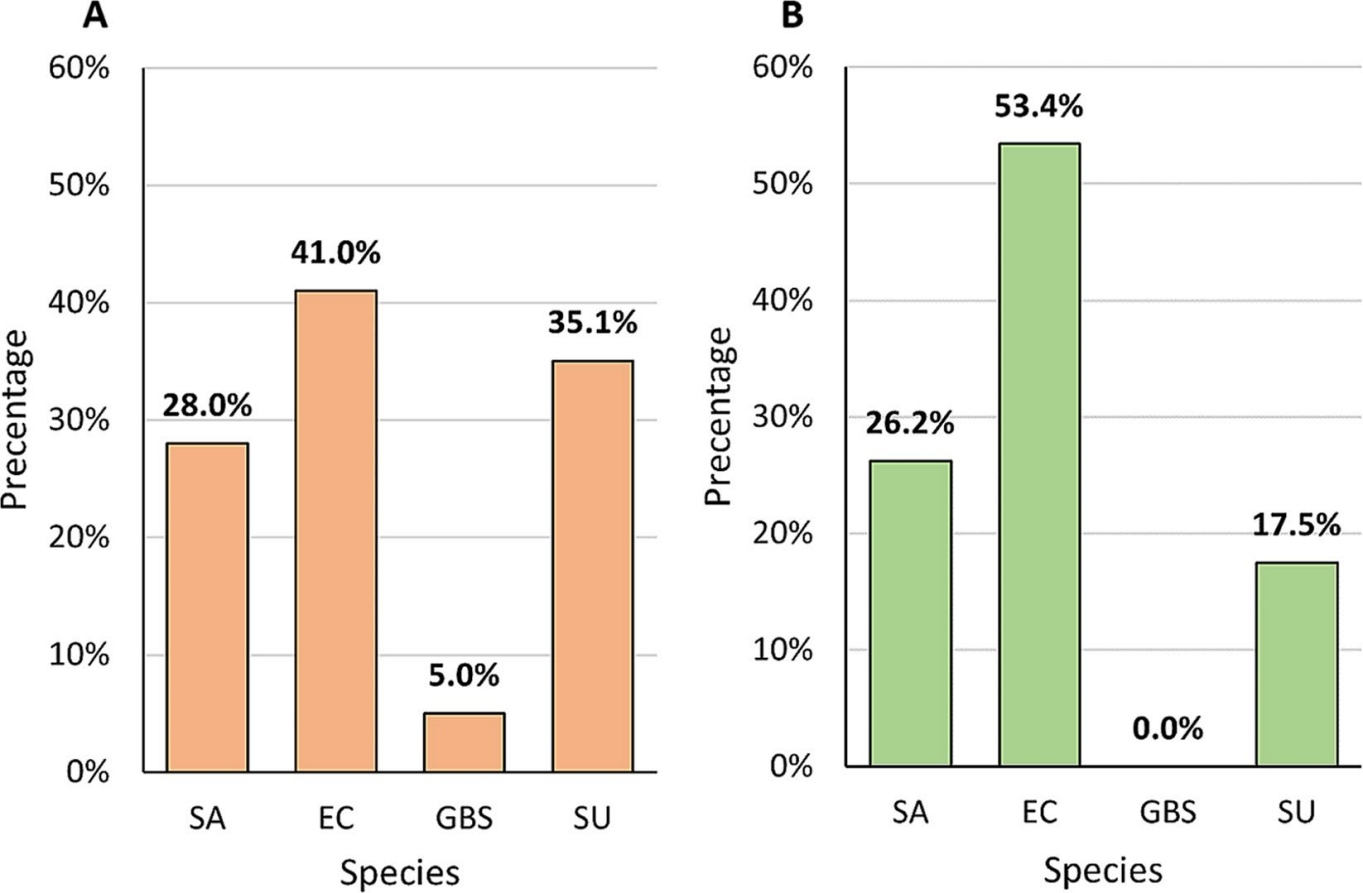
The percentage of bacterial species identified by molecular methods in bovine milk from 100 cows with clinical mastitis (**A**) and from 103 cows from the control group (**B**). Legend: SA – *Staphylococcus aureus*, EC – *Escherichia coli*, GBS – *Streptococcus agalactiae*, SU – *Streptococcus uberis*

Combined results obtained by compilation of two methods of DNA isolation (direct and pre-incubation with TSB) followed by the PCR method indicated that *E. coli* was the most frequently identified species in the pool of the CM samples examined (41.0%, n = 41). The frequency of occurrence of *S. uberis* and *S. aureus* determined by molecular methods coincided with the culture methods and the percentage was as follow: 35.1% (n = 34) for *S. uberis* and 28.0% (n = 28) for *S. aureus*. *S. agalactiae* was detected in 5 samples (5.0%) (Fig. 3A). In the control group the most common species was *E. coli* (53.4%, n = 55), followed by *S. aureus* (26.2%, n = 27) and *S. uberis* (17.5%, n = 18). *S. agalactiae* did not occur in the control group (Fig. 3B).

Results obtained by the culture methods and the molecular method demonstrated the variability in frequency of occurrence of individual species, which may result from differences in the sensitivity of the methods used, but overall the coverage (for CM and control groups) was 93.6% for *S. agalactiae*, 82.3% for *S. aureus*, 79.0% for *S. uberis* and 61.1% for *E. coli*. In-depth analysis into clinical and non-clinical samples showed that the highest coverage in CM was obtained for *S. aureus* (92.1%), followed by *S. uberis* (88.8%), *S. agalactiae* (87.1%) and *E. coli* (74.3%). In the control group the results were more divergent than for the clinical samples and the percentages obtained were as follows: the highest coverage was obtained for *S. aureus* (72.5%), for *S. uberis* 69.6%%, and for *E. coli* 48.0%. *S. agalactiae* was not found in any milk sample, so coverage reached 100.0%.

### Species co-occurrence

**Fig. 4:**
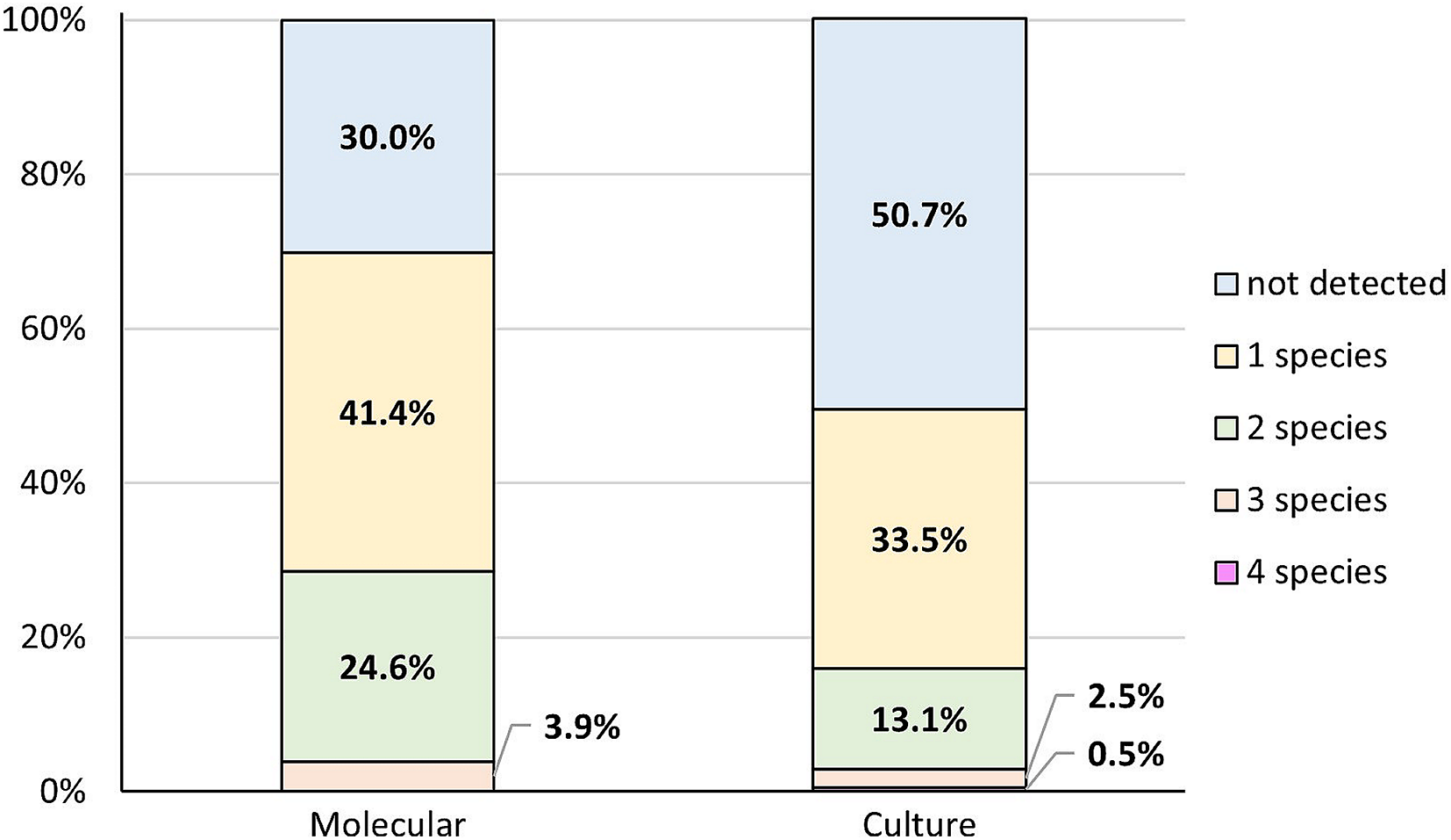
The percentage of simultaneous co-occurrence of species studied determined by the PCR method and in culture methods determined for all (N = 203) milk

The next step saw us compare the simultaneous co-occurrence of two, three and four species in the samples, both in clinical mastitis and in the control group. The most common combination found by the culture method was one species (33.5%). Two species were detected in 13.1% of samples and three species in 2.5%. Four species occurred in 0.5% of cases. In 50.7% no species were detected. In the PCR method more samples were positive, as only in 30.0% no species were detected. In 41.4% of milk samples only one species was detected. In 24.6% two species were detected and three species were detected in 3.9%. In no case were four samples detected simultaneously (Fig. 4). The differences in the combinations described were significant (p < 0.001).

**Fig. 5:**
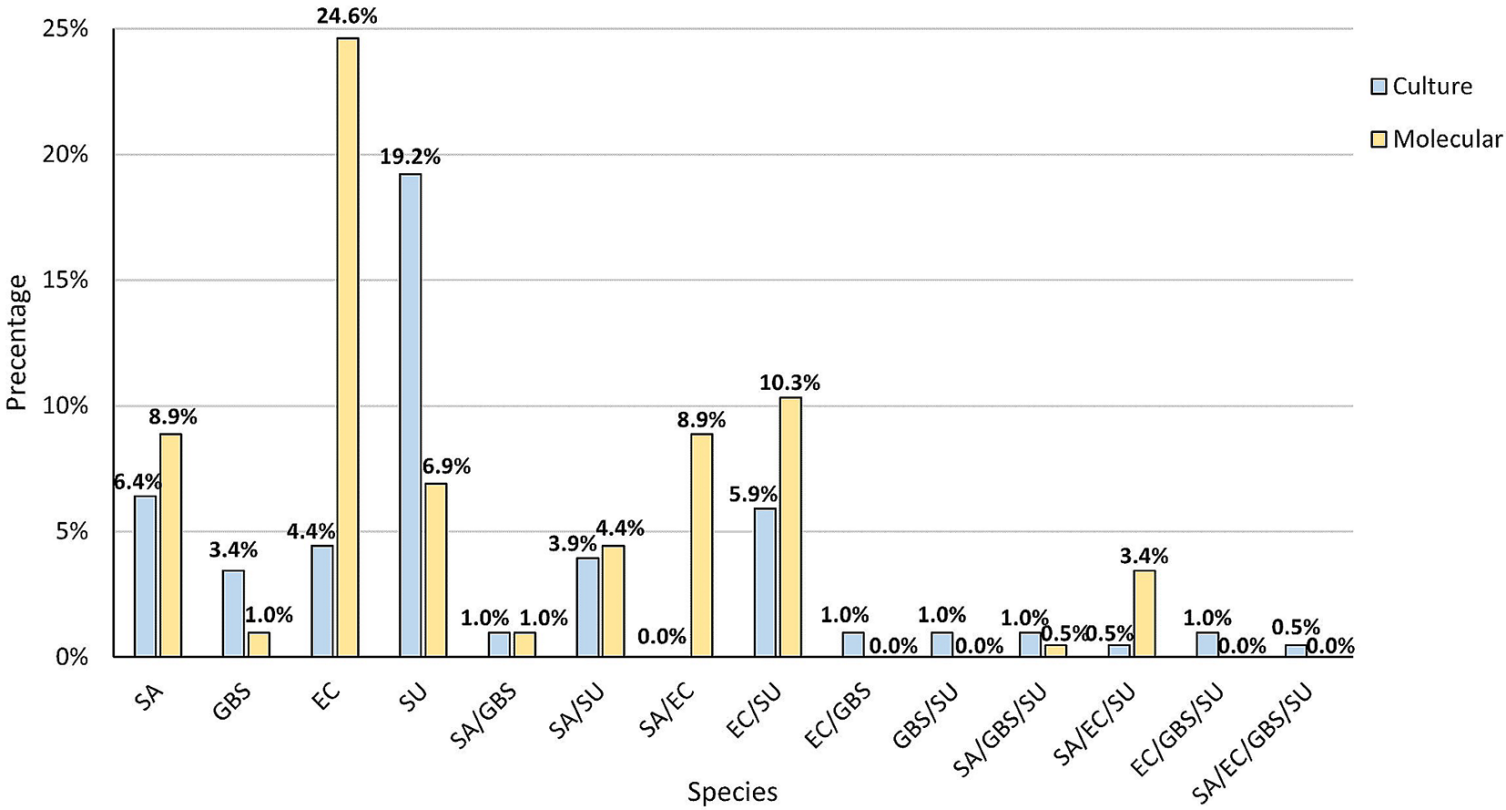
The combination of co-occurrence of the particular bacterial species of mastitis determined in the PCR method and in the culture method. Legend: SA *– S. aureus*, EC – *E. coli*, GBS – *S. agalactiae*, SU – *S. uberis*

Further in-depth analysis also demonstrated that the most frequent co-occurring species variant was *S. uberis* that simultaneously appeared with *E. coli* both according to the culture (12/203; 5.9%) and molecular (21/203; 10.3%) methods. The culture method also detected the common co-occurrence of *S. uberis* and *S. aureus* (8/203; 3.9%). The remaining combination of two, three or four co-occurred species were observed for 1 or 2 samples in the pool studied. Greater variability was noted for samples analysed by PCR in which 18/203 (8.8%) included both *S. aureus* and *E. coli*, 9/203 (4.4%) *S. aureus* and *S. uberis*, 7/203 (3.4%) were positive for the occurrence of three species *S. aureus*, *S. uberis* and *E. coli.* The simultaneous occurrence of *S. aureus* and *S. agalactiae* was shown in two samples and a variant consisting of *S. aureus*, *S. uberis*, and *S. agalactiae* was observed in one sample (Fig. 5).

### Region-dependent distribution of species

**Fig. 6:**
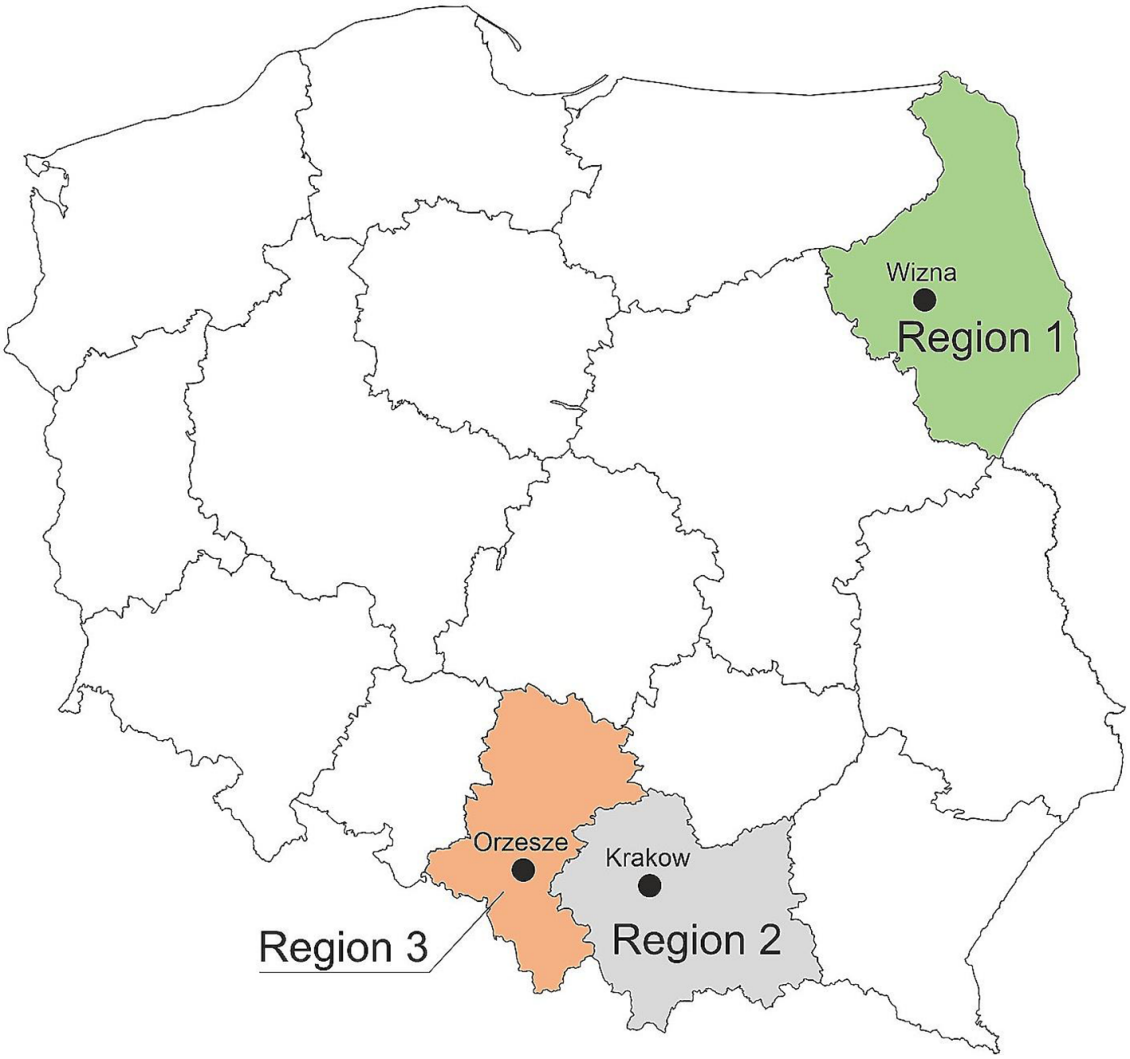
Map of Poland with marked regions and cities from which the tested milk samples came. Legend: Region 1 – the north-east, Region 2 – the south, Region 3 – the south-west

**Fig. 7:**
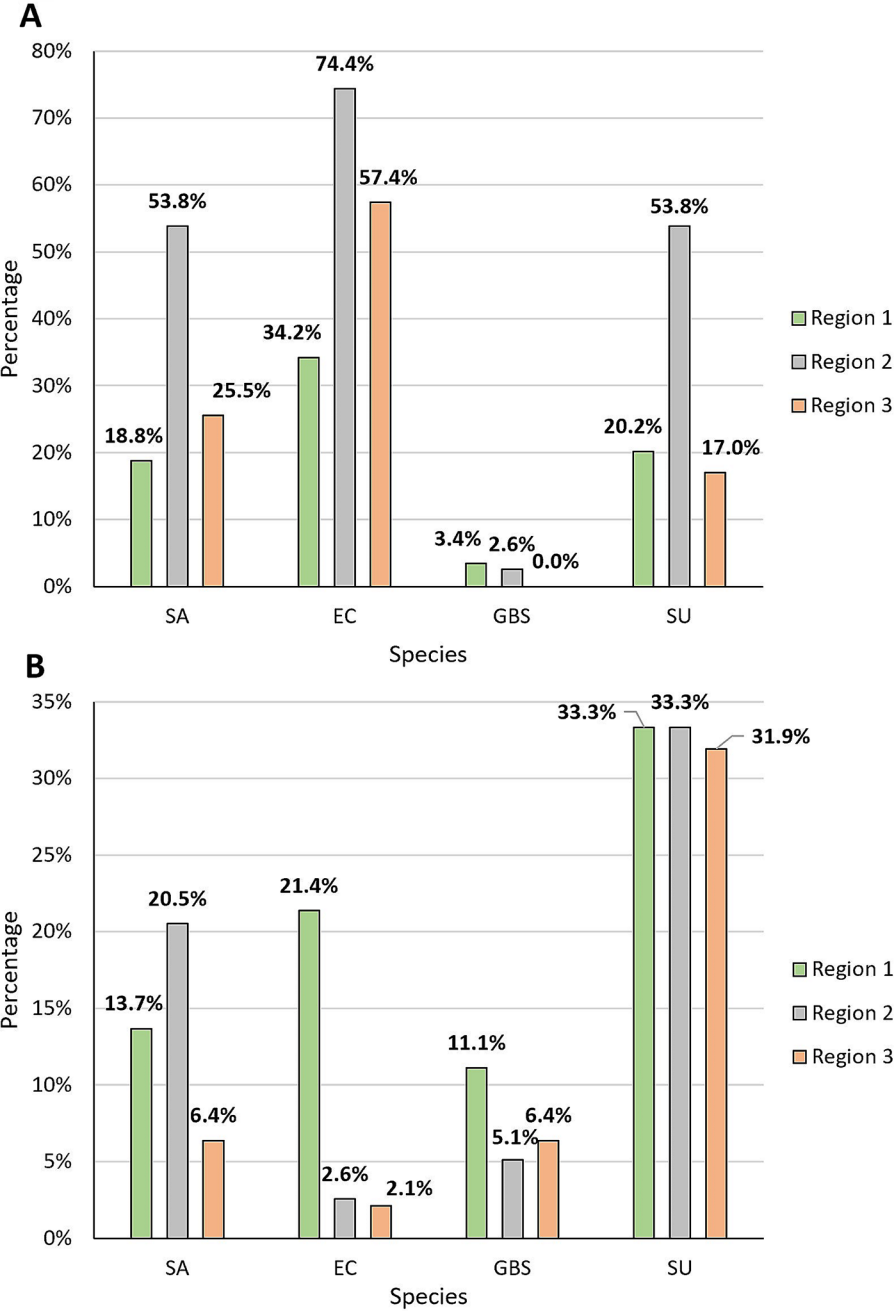
The distribution of *S. aureus* (SA), *E. coli* (EC), *S. agalactiae* (GBS), and *S. uberis* (SU) in the three regions of Poland: the north-east (Region 1), the south (Region 2) and the south-east (Region 3) determined by molecular (**A**) and culture (**B**) method

 This study also aimed to compare the composition of species between three regions of Poland: Region 1, north-eastern Poland (Podlasie); Region 2, south-western Poland (Upper Silesia); and Region 3, southern Poland (Małopolska) **(**Fig. 6**)**. Significant differences were noted for *E. coli* incidence (p < 0.001), in both the culture and molecular methods, but data obtained by the PCR method indicated that this species was the least common in north-eastern Poland, while the culture method showed that in north-eastern Poland *E. coli* was the most common species. Significant differences for the molecular method were also observed for *S. uberis* (p < 0.001) and *S. aureus* (p < 0.001). Both species were most common in southern and south-western Poland (Fig. 7).

## Discussion

Clinical mastitis (CM) is one of the most widespread diseases affecting dairy cattle and more worryingly is tends to recur, which has a significant impact on the cost of the disease in dairy cows [19–21]. The issue of mastitis is not limited to bacterial or even microbiological causes, but indubitably bacteria dominate as a an etiological factor of this disease [22]. More than half the health asymptomatic cows demonstrated mastitis streptococci (*S. uberis*, S. *dysgalactiae*, and *S. agalactiae*) [23]. It is therefore unsurprising that even 70% of the anti-microbials used in dairy farms are used to prevent and treat mastitis [24]. This problem is not only associated with financial loss for farmers and harmful effects on animals, but also pose a potential risk to public health by dint of the transmission of zoonoses and increasing multidrug bacterial resistance [25–27]. The epidemiology of bovine mastitis varies from region to region, not only due to the prevalence of different bacteria but also different sensitivity of kept breeds of cows in particular regions [28–31]. In Poland previous studies have shown that streptococcus infections are more common than staphylococcus (38.5% vs. 17.9%) and the third bacterial cause is Gram-negative bacteria such as *E. coli* (16.4%) [8], but this has also changed over the years [32].

In the culture method the most common species detected in the pool of milk samples from the clinical cases of mastitis was *S. uberis* (38.0%), which is a significant problem both in Poland and elsewhere [33]. In our previous study, the percentage of *S. uberis* in the pool of clinical samples examined was higher, reaching 44% [34]. In turn the results obtained by Dyson et al., who carried out their investigation on Australian cattle and demonstrated that *S. uberis* was present in 39.2% of the population studied [35]. Al-Harbi et al. also showed that *S. uberis* was the most common species in their study, but the frequency of occurrence was almost fourfold lower than our results [36]. This knowledge provides us with the opportunity to react to and possible prevent cases. Our results showed that the most common cause of mastitis was *S. uberis*, which knowledge is even more important because of the environmental nature of this pathogen, however some *S. uberis* strains could show a contagious nature [37]. Improving the conditions in which cows are kept, including changing the flooring, the substrate, could play a significant role [38]. The prevalence of *S. uberis* mastitis shows that the dairy industry continues to face the challenge of combatting this pathogen despite the adoption of measures to control environmental pathogens [39]. To sum up, we should still put more emphasis on management practices and improve pre- and post-milking hygiene protocols to minimise *S. uberis* mastitis.

The most frequently detected species in the pool of samples examined was *S. aureus*, which generally causes sub-clinical mastitis with a high seeding rate of infected animals and chronic recurrent infections. The virulence of *S. aureus* results from its ability to produce biofilm, toxins and various enzymes that lead to cell damage in the host and allows the bacteria to initiate the host invasion [40, 41]. Our studies confirmed the presence of *S. aureus* in 22% (culture method) and in 28% (PCR method) of CM samples. Polish studies carried out by Lassa et al. supported our results in which *S. aureus* accounted for 22.9% [8]. The percentages yielded in this study also corresponded with the results in a large-scale investigation carried out by Liu et al., who demonstrated that *S. aureus*, depending on the farm examined, reached up to 24.2% [42]. The prevalence of *S. aureus* is influenced by many factors, starting from sanitary and hygienic conditions, local strains/genotypes, cattle breeds, and bedding, which differs significantly between countries [43, 44]. American studies showed that *S. aureus* was infrequent (2.8%) [45]. Unfortunately, in Poland, despite the constant improvement of sanitary and hygienic conditions, breeders’ awareness of mastitis prevention is insufficient. Constant screening and monitoring for *S. aureus* are vital because, even after many years of studying *S. aureus*, no effective therapy has been developed due to its rapid genetic variability [43, 46]. It is therefore unsurprising that the drug-resistance of *S. aureus* prevails [47].

*E. coli* was third the most common CM species and is classified as an environmental pathogen. The frequency of *E. coli* infections increase during summer. This is explained by the seasonal rise in temperature and humidity. As a result of heat stress, the cows’ immunity also diminished, which may lead to an increase in the risk of infection [48]. In our study, the results of the culture method indicated that *E. coli* was present in 21% of pool of samples examined. In a previous study, on a smaller number of samples, *E. coli* was present in 18.2% of the milk examined [34]. Studies on Nepalese cattle showed the incidence of mastitis caused by *E. coli* was over 16% of samples examined [49]. Comparable results were obtained in France [50]. In Poland the incidence of mastitis caused by *E. coli* in 2020 was shown at only 2.7% [51]. The differences may result from the number of samples included in Krukowski et al.’s study, as they included over 38.000 milk samples, obtained from clinical and subclinical cases of mastitis. In our study percentage of *E. coli* was determined only for clinical cases. Infection caused by *E. coli* may be the result of a number of factors. Some strains of *E. coli* acquire specific virulence factors (VFs), which may enable them to infect the mammary glands and multiply in milk [52, 53]. The host’s immunological system, however, demonstrates an ability to protect itself from *E. coli* infection thanks to components of innate immunity components, such as anti-microbial peptides, lysozyme, lactoferrin and other complements [54, 55]. Some strains of *E. coli* associated with mastitis also evolved mechanisms that allow them to compete with other bacteria co-infecting the gland [48, 56], which may explain that coinfection with *E. coli* is the most common in our study.

*S. agalactiae*, which is responsible for cases of contagious mastitis, was the fourth most common species identified in the CM milk sample studied. It was recognised as a highly contagious obligate bacterium of the bovine mammary gland, which tends to be incapable of surviving for long periods outside the mammary gland [57]. The latest research, however, confirms its ability to survive in extra-mammary sources [58]. In mammary glands *S. agalactiae* also has the ability to survive by dint of forming biofilms [59] and as a variety of other bacteria becomes increasingly resistant to appropriate treatment [60]. The prevalence of *S. agalactiae* in bovine mastitis due to the introduction of the mastitis control programme [61, 62] has dwindled in recent decades. In the 1980s, however, it was the main cause of mastitis, being responsible for almost 50% of cases [36, 57, 63]. The decrease in the incidence of *S. agalactiae* detected in mastitis milk samples was also noted in Poland [64]. In our study *S. agalactiae* was present in 19% of CM milk and the results corresponded with other Polish studies carried out by Sztachańska *at al.*, who detected *S. agalactiae* in 15% of samples examined [65]. Malinowski et al. demonstrated that the presence of this bacterium varied between 2% and 25% on Polish farms [32].

In the control group the diversity in the combination bacterial species was greater than in the CM group. Whereas in the CM group 5 species were detected, in the milk from cows with no clinical signs of mastitis 8 species were detected. The species identified, especially these classified as environmental, e.g. *Viridians group Streptococcus*, may indicate that contamination of the samples with the skin the microbiota and may result from failure to follow the rules of sterile milk sampling. The high frequency of the occurrence of these species, as high as 1/3 of pool studied, in spite of our observing aseptic rules during the collection of biological material, indicates the need for even greater care when collecting material for examination. We are also aware that study material delivery on dry ice under deep-freeze conditions and storage at -80˚C could have an impact on the bacterial composition and the bacterial number.

The choice of suitable diagnostic methods significantly influences the reliability of the results and, consequently, epidemiological data. Many studies rely solely on culture techniques and biochemical tests [8, 32, 36, 65]. Our research and other publications [66–69] confirm the necessity of complementing these methods with molecular techniques such as PCR or real-time PCR reactions. However, it should be considered that the higher sensibility of PCR can be related to the DNA amplification of a not significant number of bacterial colonies in milk (that cannot be considered as responsible for mastitis), or dead bacterial cells. However, in our study, for some milk samples, we observed that while positive results were obtained using culture methods, the result was negative with use of PCR amplification. Nevertheless, this observation shows that there are exceptions to the above assumption, for example due to the presence of PCR inhibitors. In this study, the use of molecular methods was intended to confirm the results obtained using culture methods and to additionally complement them. However, in order to achieve this goal, it was necessary to use 18 h of preincubation. In turn, the limitations of culture methods in relation to molecular methods are a topic discussed in many publications which emphasize, for example, the influence of inhibitory products of bacterial metabolism, the presence of dominant species, which influences the inhibition of the growth of subordinate species, unfavorable conditions during sample transport as factors that may reduce the viability of bacteria, which in turn may contribute to obtaining a false negative result in culture despite the occurrence of mastitis symptoms. In such cases, the use of molecular methods allows obtaining a positive result and complements culture methods. The crucial preliminary step before amplification is the extraction of bacterial DNA. In certain publications the standard isolation procedures [66, 67, 69–71] are described as involving direct extraction from milk samples by use of commercial kits. In our research, as well as employing the standard approach, we also evaluated the effect of an additional 18-hour pre-incubation of milk samples in TSB on the sensitivity of the detection of micro-organisms in the PCR reaction. The obtained results suggest the necessity of this step, as it led to an increased detection rate of *Streptococcus uberis, Staphylococcus aureus, Escherichia coli* and *Streptococcus agalactiae* by 18.1%, 11%, 9% and 1%, respectively in comparison to direct isolation. For *S. aureus* and *S. uberis* the results obtained after pre-incubation also align with those achieved through culture methods. A similar approach was described by Riffon et al. [72], who aimed of the research was to develop a sensitive and cheap PCR reaction enabling the detection of six main pathogens that cause mastitis (*Escherichia coli, Staphylococcus aureus, Streptococcus agalactiae, Streptococcus dysgalactiae, Streptococcus parauberis and Streptococcus uberis*), however the study was conducted, in opposition to ours, only on reference well characterized strains, with which sterile milk was infected, The developed PCR method, however, was not used to identify pathogens from milk samples collected from cows (with or without mastitis) or to compare the results with culture methods. Ding *et al’s* [73] also focused on the need to pre-incubate milk samples before bacterial DNA isolation and for this purpose five different liquid media were tested (alkaline peptone water (APW), Brain Heart Infusion broth (BHI), Luria-Bertani broth (LB), TSB and peptone water (PW)). The highest maximum population density of mastitis pathogens was achieved in LB, BHI and TSB, but these tests were conducted only for *S. aureus*, *L. monocytogenes* and *Salmonella* spp. In our research the use of enrichment TSB medium not only increased the concentration of micro-organisms but probably also enabled the dilution of PCR inhibitors contained in milk [74], e.g. the concentration of calcium or plasmin [75]. All of this may explain the observed quantitative differences in bacterial detection between enrichment and non-enrichment isolation. Pre-incubation in TSB, however, may introduce certain limitations, such as the potential for undesirable bacteria to grow and compete for nutrients with the targeted bacteria, or domination by the culture of fast-growing bacteria, which may mask the presence of slower-growing or more delicate target bacteria [73]. The identification of micro-organisms by PCR is difficult and has a direct impact on obtaining false negative results.

The above limitations of pre-incubation in TSB may explain our study’s lower percentage of positive results for *S. agalactiae* and *S. uberis* species after PCR than culture methods by 13% and 20.5% respectively (4% in the clinical group and 16.5% in the non-clinical group). The false-negative results concerned only streptococci, highlighting the necessity in future studies of employing a different pre-incubation medium that will be more selective towards streptococci. With regard to the remaining species, *S. aureus* and *E. coli*, molecular methods yielded a significantly higher number of positive results than culture methods, by 27.3% and 67.6% respectively (total values for the clinical and non-clinical groups). Higher PCR sensitivity in relation to culture for *S. aureus* or *E. coli* was also obtained in Nyman et al.’s [76], Graber et al.’s [77]and Koskinen et al.’s [78] research. In Koskinen’s work especially large discrepancies between these methods were observed. The use of PCR yielded an additional 53 and 68 positive samples for *S. aureus* and *E. coli* respectively, which were negative in culture. A significantly (p = 0.002) higher prevalence of this species in comparison with culture method may, however, be the result of sensitivity of the primers used and the PCR conditions that detect the remaining DNA in the sample rather than the DNA from the etiological factor that caused mastitis in individual cases. We hypothesise that the differences in results between methods may be caused by autolysis of *E. coli*, the presence of dead or damage bacterial cells in milk samples, which are unable to grow on the solid agar plate, but they still have bacterial DNA, detectable by molecular methods. These methods may also detect remaining DNA in samples, for example, after previous infections or as a contamination, hence not necessarily DNA from etiological factors causing mastitis in individual case.

## Conclusions

As bovine mastitis constitutes to cause severe worldwide economic and epidemiologic issues, constant monitoring and management are crucial in order to curb this threat to the health and life of cattle. We are aware that a survey of more herds from more voivodeships would deliver even more informative results, but the percentage values obtained in our study correspond with the prevalence noted by other researchers. We therefore believe that further investigation would only confirm our observation. We believe that our results would increase and update epidemiological knowledge about mastitis among cows in three regions of Poland, including the Region 3 with the biggest concentration of cows in the country, and may encourage for further investigation including remaining Voivodeship as a relevant data is missing.

## Methods

The investigation included 203 milk samples, obtained from 2 to 6 years old Holstein Friesian cattle, divided into two groups: (I) the study group, which included 100 milk samples from cattle with clinical signs of mastitis (CM) displaying the following symptoms: swelling, heat, hardness, redness or pain of the udder; watery appearance, flakes, clots or pus in milk; increased body temperature or lack of appetite, and (II) the control group, which included 103 milk samples from cattle with no clinical symptoms of mastitis. The number of samples was equal to the number of cows tested. From cows with active mastitis, a milk sample was collected only from the inflamed quarter, for healthy cows, milk from four quarters was pooled to one sample. According to the guidelines of National Mastitis Council (NMC) five or more colonies of an environmental species were considered as an infection while three or more species in one sample was treated as a contamination [79].

Milk samples were collected from udder quarters in the routine course of the submission of milk from different regions in Poland: Omniwet Veterinary Clinic, Orzesze (south-western Poland) (n = 47), Egida Veterinary Clinic, Wizna (north-eastern Poland) (n = 117), and University Centre for Veterinary Medicine Institute of Veterinary Sciences, Krakow (southern Poland) (n = 39). Cows included in the investigation came from different cowsheds, both stationary and free-standing cowsheds. The health status of the animals had been determined on the basis of physical parameters, such as the absence of edema or redness of the udder, as well as biochemical parameters by determining the number of somatic cells by California Mastitis Test (CMT) (animals with somatic cells > 100.000/ml and with clinical signs were classified as sick, whereas the lack of symptoms was a basis for classifying the animals as healthy). The herds studied were assessed by the Polish Federation of Cattle Breeders and Milk Producers, which requires monthly reports of milk parameters, such as the number of somatic cells per cow milked. According to the 1st local Ethical Committee at the Jagiellonian University Medical College in Cracow, Poland consent for the research project was not required, as the study has not direct involvement of animals. Milk samples that were delivered to the Department of Microbiology on dry ice under deep-freeze conditions were stored at -80˚C for further analyses.

### Bacterial culture and phenotypic identification

100 µl of each milk sample was inoculated separately on each type of medium: on Columbia sheep blood agar (BioMaxima) [80] and CHROMagar™ Mastitis (BioMaxima) [81], and then cultured at 37˚C under aerobic conditions for 18 h. Individual bacterial colonies that grew on Columbia Agar and CHROMagar™ Mastitis and macroscopically corresponded to the pathogenic bacteria that constitute the most common etiological agent of mastitis were then inoculated onto selective media such as MacConkey agar (Graso) selective and differential medium for the identification of Gram-negative rods, Chapman’s (Graso) selective and differential medium for the identification of *S. aureus* [82], Granada chromogenic medium (Beckton Dickinson) [83] for the identification of *S. agalactiae* and esculin medium (Bio Maxima S. A.) [78] for the culture and differentiation of *S. uberis* from *S. agalactiae*. The culture was carried out at 37˚C under aerobic or microaerophilic conditions for 18 h. Isolated bacterial colonies were also suspended in 0.9% NaCl and spotted on glass slides until dry. Microscopic smears were then stained with the Gram method using standard dyes (STAMAR) and observed under a light microscope (OLYMPUS CX21) at 100x magnification using oil immersion (MERCK). In order to confirm cultivated bacterial species, some biochemical tests, such as the catalase test using 9% hydrogen peroxide (ALCHEM), the coagulase test using rabbit plasma (BIOMED) and the latex agglutination test using Streptococcal Grouping Kit (OXOID) [84] to confirm the species of the tested microorganisms were performed.

On the basis of the results obtained we also attempted to compare Columbia blood agar and Chromagar mastitis in precise identification of the bacterial species most common in mastitis.

### Molecular identification

Species were identified by use of polymerase chain reaction (PCR) for four bacterial species: *S. uberis*, *S. agalactiae*, *S. aureus*, and *E. coli*, since they are the most common etiological factors in bovine mastitis.

The first step involved the original milk samples being thawed, intensively and pulse vortexed, before being transferred in volumes of 500 µl to sterile, screw-cap tubes with glass beads (Sigma-Aldrich). 2 isolation approaches were then performed, the first involving the isolation of bacterial DNA directly from the milk sample, and the second, involving the pre-incubation of the milk samples with tryptone-soy broth (TSB, Becton Dickinson) in 1:1 proportion for 18 h at 37˚C in aerobic conditions and before being subjected to bacterial isolation. As well as subjecting each sample to the first or second approach, 20 µl of lysozyme (50 mg/µl, Sigma-Aldrich), 15 µl of lysostaphin (1.0 mg/ml, A&A Biotechnology), and 5 µl of mutanolysin (10U/µl, Sigma-Aldrich) was added and the tubes were homogenised in a FastPrep device (Thermo Savant) for 1 min. After incubation for 30 min at 37˚C and vortexing, the samples were transferred to new tubes included in the GeneProof automated isolation kit (Imogena), 20 µl of proteinase was added and they were subjected to automatic isolation on CroBEE apparatus. The DNA isolates obtained were stored at -20 °C for further analysis.

The next step saw the amplification of isolated DNA by the use of four pairs of primers [58, 71, 72] (Table 1) dedicated to the detection of *S. uberis*, *S. agalactiae*, *S. aureus*, and *E. coli* in the material studied.

**Table 1:** List of primers used in the study and predicted sizes of PCR products for the amplification of *E. coli*, S. aureus, *S. agalactiae* and *S. uberis*

Studied species	Name	Primers	Source	Product size (bp)
*Escherichia coli*	EC_F	F: GGTAACGTTTCTACCGCAGAGTTG	[66]	460
	EC_R	R: CAGGGTTGGTACACTGTCATTACG		
*Staphylococcus aureus*	SA_F	F: AATCTTTGTCGGTACACGATATTCTTCACG	[85]	100
	SA_R	R: CGTAATGAGATTTCAGTAGATAATACAACA		
*Streptococcus agalactiae*	GBS_F	F: CGATTCTCTCAGCTTTGTTA	[86]	780
	GBS_R	R: AAGAAATCTCTTGTGCGGAT		
*Streptococcus uberis*	GBS_F	F: TCGCGGTATTGAAAAAGCAACAT	[66]	400
	GBS_R	R: TGCAATAATGAGAAGGGGACGAC		

For each bacterial species, the PCR reaction conditions were optimized including the temperature range, oligonucleotide concentrations, DNA template volume [µl] and the number of amplification cycles. As a positive control, both in standardisation and amplification the DNA of reference strains (ATCC 25,923 for *S. aureus*, ATCC 25,922 for *E. coli*, ATCC 21,403 for *S. agalactiae*) was used. Each time a reaction mixture consisting of sterile water free of Dnase and Rnase as a negative control was included. On the basis of the experiments conducted, the composition of the reaction mixtures for the following pairs of species *S. agalactiae* and *S. aureus* (pair 1), and *E. coli* and *S. uberis* (pair 2) were determined separately.

For *S. agalactiae* and *S. aureus* the following reagents were used: 5.0 µl of PCR Master Mix (A&A Biotechnology), 0.3 µl of GBS_F and GBS_R primers (10 µM, Genomed S. A.), 0.4 µl of SA_F and SA_R primers (10 µM, Genomed S. A.), 1.5 µl of studied DNA and 2.1 µl of DNAase free water (A&A Biotechnology). For *E. coli* and *S. uberis* reagents included: 5.0 µl of PCR Master Mix (A&A Biotechnology), 0.3 µl of EC_F and EC_R primers (10 µM, Genomed S. A.), 0.3 µl of SU_F and SU_R primers (10 µM, Genomed S. A.), 1.5 µl of the DNA studied and 2.3 µl of DNAase free water (A&A Biotechnology). The amplification procedure was carried out using the T100 Thermal Cycler (BioRad) according to the programme: preliminary denaturation at 92° C for 3 min prior to 40 repeated cycles: 1 min denaturation at 92° C, 1 min introduction of primers at 56° C and 1 min elongation at 72° C finished with 3-min final extension in 72° C. The products of amplifications were then separated on 2% agarose gel (Prona ABO) in 1× TBE buffer (Sigma-Aldrich) with the addition of ethidium bromide (Sigma-Aldrich) for 60 min at voltage 100 V. The results obtained from electrophoretic separation were analyzed in Gel Doc System FastGene (FAS-DIGI PRO).

### Statistical analysis

Statistical analyses were performed using IBM SPSS Statistics version 29. The relationships between categorical variables were assessed using the Pearson χ2 test and Fisher’s exact test. The results were presented as numbers and percentages. Statistical significance was defined as p < 0.05 for all tests.

## Data Availability

The data underlying this article will be shared on reasonable request to the corresponding author.

## Abbreviations

CM: Clinical Mastitis
GBS: Group B Streptococcus
NASM: Non–aureus staphylococci and mammaliicocci
SCC: Somatic Cell Count
NAATs: Nucleic Acid Amplification Techniques
LFA: Lateral Flow Assay
SV: Viridans Group Streptococci
PCR: Polymerase Chain Reaction
TSB: Tryptone–Soy Broth
TBE: Tris–Borate–EDTA buffer
